# The Mediating Effect of Socioeconomic Status and Persistent Poverty on Racial and Ethnic Disparities in Pediatric Cancer Mortality in the United States

**DOI:** 10.1002/cam4.71638

**Published:** 2026-02-22

**Authors:** Josiane Kabayundo, Apu Das, Cheng Zheng, Emma Hymel, Krishtee Napit, Shinobu Watanabe‐Galloway

**Affiliations:** ^1^ Department of Epidemiology, College of Public Health University of Nebraska Medical Center Nebraska USA; ^2^ Department of Biostatistics, College of Public Health University of Nebraska Medical Center Nebraska USA

**Keywords:** cancer, mediation analysis, mortality, pediatric, persistent poverty, racial and ethnic disparities, socioeconomic status

## Abstract

**Background:**

Socioeconomic (SE) factors have been shown to mediate racial and ethnic disparities in adult cancer mortality. However, evidence in pediatric cancer is limited, especially at finer geographic levels.

**Objective:**

Evaluate the mediating effect of socioeconomic status (SES) and persistent poverty in racial and ethnic disparities in childhood cancer mortality using census tract‐level data.

**Methods:**

Data were obtained from the 2006–2020 SEER Incidence Data with Census Tract Attributes Database and included children 0–19 years. Neighborhood‐level SES was measured using a composite variable with five categories. Census tracts were classified as persistently poor if 20% or more of the population lived below the poverty level for 30 years. Cause‐specific Cox proportional‐hazard models were used to examine the association of race and ethnicity with cancer mortality, adjusting for age, gender, stage at diagnosis, cancer type, and rurality. A weighted approach mediation analysis was performed in R4.1.3.

**Results:**

Among 96,665 included cases, 19.95% of children lived in the lowest SES quintile, and 12.23% lived in areas with persistent poverty. The distribution of SES and persistent poverty varied across race and ethnicity. Specifically, 9.79% of White children lived in the lowest SES neighborhoods compared to 33.77% of Black and 32.48% of Hispanic children. In addition, 4.83% of White children lived in persistent poverty compared to 21.38% of Black and 21.30% of Hispanic children. Compared to White children, the risk of cancer death was higher among Black (aHR: 1.53; 95% CI: 1.45–1.62), Hispanic (aHR: 1.17; 95% CI: 1.12–1.22), and Asian (aHR: 1.24; 95% 1.15–1.33) children. The proportion mediated by SES was 13.50% for Black, 29.3% for Hispanic, and 37.58% among AI/AN children. The proportion mediated by persistent poverty was 6% for Black, 12.79% for Hispanic, and 17.47% for AI/AN children.

**Conclusion:**

SES and persistent poverty significantly contributed to racial and ethnic differences in pediatric cancer mortality.

## Graphical abstract

Socioeconomic status and persistent poverty explain racial and ethnic disparities in pediatric cancer mortality, highlighting the need for interventions addressing socioeconomic factors both at the individual and neighborhood level.

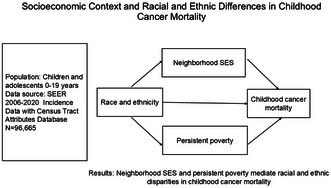

## 1 Background

Pediatric cancer remains a critical public health issue in the United States. In 2024, an estimated 9620 children (ages 0–14 years) and 5290 adolescents (ages 15–19 years) were diagnosed with cancer [1]. Moreover, cancer remains the second leading cause of death after accidents among children aged 0–14 years [1]. It was estimated that 1040 children and 550 adolescents died from cancer in 2024 [1]. Recent discoveries in pediatric cancer diagnosis and treatment, including risk classification, genetic‐based classification of tumors, combination of therapies, and targeted therapy, have decreased pediatric cancer mortality and improved survival [2, 3, 4]. Since 1970, pediatric cancer deaths have decreased by 70% among children 0–14 years and by 63% among children 15–19 [1]. Moreover, the five‐year survival rate increased from 63% in 1970 to over 85% in 2016 [1]. However, significant racial, ethnic, and socioeconomic disparities in pediatric cancer outcomes persist to this date [5, 6, 7].

Among neighborhood‐level socioeconomic indicators, socioeconomic status (SES) and persistent poverty play key roles in childhood cancer survival. Cross‐sectional neighborhood SES at diagnosis has been associated with later stage at diagnosis, reduced survival, and higher mortality in children with cancer [3, 5, 6, 7]. In contrast, persistent poverty reflects the entrenched and structural nature of disadvantages [8, 9]. A neighborhood is defined as persistently poor when at least 20% or more of the residents have lived below the federal poverty level for the past 30 years [8]. Children living in persistent poverty neighborhoods face systemic barriers such as greater distance from treatment centers, limited access to resources, and structural disadvantage that translate to higher cancer incidence, increased likelihood of late‐stage diagnosis, poorer survival, and higher cause‐specific mortality [9, 10, 11, 12, 13].

Importantly, individuals from minority racial and ethnic groups are disproportionately concentrated in low SES and persistently poor neighborhoods. A recent study showed that 20% of children diagnosed with cancer annually live in poverty [14]. Moreover, neighborhoods with persistent poverty experience 12% higher cancer mortality than non‐persistent poverty neighborhoods [9, 15]. Despite the availability of evidence indicating racial, ethnic, and socioeconomic pediatric cancer mortality disparities and the correlation between race and ethnicity and socioeconomic factors, little research has investigated the mechanisms by which SES and persistent poverty contribute to racial and ethnic survival disparities in pediatric cancer. A conceptual model proposed by Wolfson in 2021 suggests that to address systemic disparities in cancer mortality and survival, the mechanism through which socioeconomic factors affect cancer outcomes should be understood [14].

Mediation analysis offers a powerful framework for addressing this critical gap. By decomposing the relationship between race and ethnicity and pediatric cancer survival into direct pathways and indirect pathways (through SES and persistent poverty), mediation analysis can clarify the extent to which long‐term neighborhood disadvantages contribute toward disparities. Such evidence is vital for guiding policy, advancing equity‐focused interventions, and identifying structural level factors to improve outcomes for the most disadvantaged children with cancer. Studies conducted among adults indicate that socioeconomic factors may explain observed racial and ethnic survival disparities, specifically by mediating the relationship between race and ethnicity and survival outcomes [16, 17, 18, 19]. Among pediatric cancer patients, although previous studies show that late‐stage diagnosis, tumor characteristics, and limited access to healthcare could explain racial and ethnic disparities in childhood cancers [20, 21, 22], there is a lack of studies examining the role of socioeconomic factors in racial and ethnic disparities in pediatric cancer mortality. To date, only one study has examined neighborhood SES as a mediator [23], and none has addressed the mediating role of persistent poverty. Therefore, our study uses mediation analysis to evaluate the role of SES and persistent poverty in racial and ethnic cause‐specific mortality in childhood cancer.

Building on previous work by Kehm, et al., which used data on children diagnosed between 2000 and 2011 [23], our study extends the analysis of childhood cancer survival to cases diagnosed between 2006 and 2020. Unlike prior research that focused on neighborhood SES alone, this study is the first to evaluate whether persistent poverty mediates racial and ethnic disparities in pediatric cancer mortality. By integrating both cross‐sectional and long‐term indicators of socioeconomic disadvantages, we provide a novel contribution to understanding the structural roots of survival disparities.

## 2 Methods

### 2.1 Data Source and Case Selection

**FIGURE 1:**
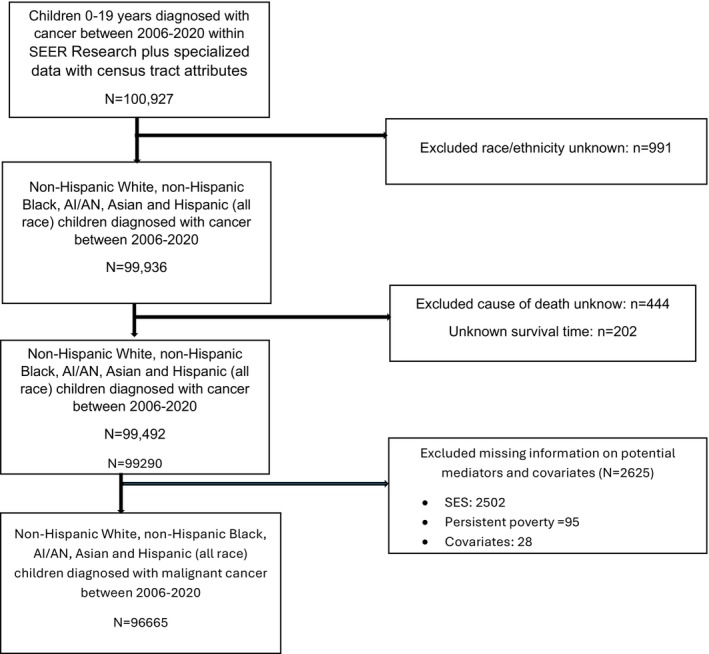
Study participants selection.

We used Surveillance, Epidemiology, and End Results (SEER) Research Plus Specialized Incidence Data with Census Tract Attributes Database (22 registries excluding AK, IL, and MA), 2006–2020 data. The SEER is a program of the National Cancer Institute. It provides information on cancer incidence and survival from population‐based cancer registries covering approximately 48% of the US population (42.0% of White, 44.7% of African American, 66.3% of Hispanic, 59.9% of American Indians and Alaska Natives, 70.7% of Asian, and 70.3% of Hawaiian/Pacific Islanders) [24]. We used a case‐listing approach to identify eligible individuals. We included children diagnosed with malignant cancer at ages 0–19 between 2006 and 2020. We excluded children with missing information on the variables of interest (Figure 1).

### 2.2 Variables and Measurements

#### 2.2.1 Exposure

The exposure variable is race and ethnicity, classified as Non‐Hispanic White (herein referred to as White), Non‐Hispanic Black (herein referred to as Black), Non‐Hispanic Asian/Pacific Islander (API) (herein referred to as Asian), Non‐Hispanic American Indian or Alaska Native (AI/AN) (herein referred to as American Indian/Alaska Native), and Hispanic (all races). SEER recommends this combined classification [8]. In SEER, races are extracted from medical records, which are either self‐reported or inferred from the provider [18]. Ethnicity is determined through self‐report, medical record, or by a computer algorithm that identifies surnames and maiden names of Spanish origin [23].

#### 2.2.2 Outcome

The outcome of interest was cancer‐specific death, which was determined using the International Disease Classification of Diseases Version 10 [25]. The follow‐up of included participants was from the date of pediatric malignant cancer diagnosis to the date of death or the end of the study period. Individuals who died from other causes or were alive at the end of the study period were censored.

#### 2.2.3 Mediators

Neighborhood‐level SES and persistent poverty were assessed separately as potential mediators. SES is a composite variable available in SEER. The variable is measured at the neighborhood level using the residential address at the date of the cancer diagnosis. The SES index was pre‐calculated through a factor analysis using seven indicators of neighborhood socioeconomic status, including the proportion employed in working‐class occupations, the proportion aged 16 years or older and unemployed, education index, median household income, proportion below the 200% poverty level, median rent, and median house value obtained from the US Census and the American Community Survey [26, 27]. The index is a 5‐level variable categorized into quintiles, with quintile 1 being the lowest SES quintile and quintile five the highest [8]. Persistent poverty is also available within SEER and was classified as persistent poverty or non‐persistent poverty. A census tract is designated as persistent poverty if 20% or more of the population lived below the poverty level for 30 years based on 1990 and 2000 decennial censuses and 2007–2011 and 2015–2019 American Community Survey five‐year estimates [8].

#### 2.2.4 Covariates

Sociodemographic factors (age at diagnosis and gender), clinical factors (cancer type and stage), and rural–urban residence were included as covariates. Cancer types were classified using the International Classification of Childhood Cancer Third Edition (ICCC‐3) [28]. Rural–urban residence at the county level was classified using the 2013 Rural–Urban Continuum Code (RUCC). Patients in counties with codes 1–3 were classified as urban, and those in counties with codes 4–9 as rural.

### 2.3 Statistical Analysis

Chi‐square tests were used to assess differences in demographic characteristics of study participants by race and ethnicity. Cause‐specific Cox proportional hazard models were used to examine the association between race and ethnicity or each mediator with cancer mortality, adjusting for age, gender, rurality, cancer type, and stage at diagnosis. In addition, a stratified analysis by cancer type was performed based on prior knowledge of survival differences by cancer type. Interaction terms were added to the model to test for the interaction between race and ethnicity and each mediator (one at a time, added separately to the model, adjusting for covariates). The proportional hazards assumptions were tested using Schoenfeld residuals. To estimate the mediating effect of SES and persistent poverty, a weighting approach was performed using the R4.1.3 mediation package (R for Statistical Computing). The weighting approach computes the mediation weights based on the mediation model to estimate the direct and indirect effects of interests and reduce the possibility of modeling bias [29, 30]. The weighting approach is more flexible than traditional approaches. Moreover, unlike traditional mediation approaches that use regression‐based decomposition of effects, the weighting approach does not combine parameter estimates from the mediator and outcome models [30]. The total effect was estimated using the product of the NDE and NIE. A significant NIE indicated a significant mediating effect of mediators [31]. The proportion mediated was calculated using the log (NIE)/log (TE). We performed separate analyses for each mediator; in addition, a separate model was constructed for the three most common cancer types: leukemia, CNS tumors, and lymphoma.

## 3 Results

### 3.1 Sample Description

**FIGURE 2:**
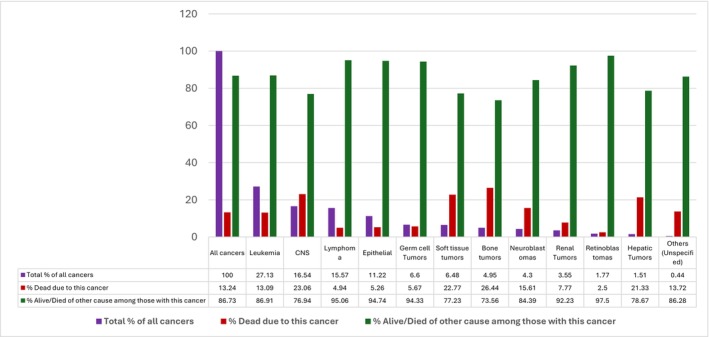
Death status by different cancer types.

Descriptive characteristics by race and ethnicity are provided in Table 1. Of 100,927 identified cases, 96,665 with complete data on variables of interest were included (Figure 1). Among included children, 48.16% were White, 10.29% were Black, 33.90% were Hispanic, 7.12% were Asian, and 0.53% were AI/AN. 53.45% were male and 46.55% were female. Most cases were between 15 and 19 years (32.10%), followed by 1–4 years (23.61%), 10–14 years (2.14%), 5–9 years (17.45%), and less than 1 year old (6.69%). The most common cancer types were leukemia (27.13%), CNS tumors (16.54%), lymphoma (15.57%), and epithelial tumors (11.12%). Overall, 19.95% of children lived in neighborhoods classified in the lowest SES quintile (quintile 1), and 12.23% lived in areas with persistent poverty. The distribution of SES and persistent poverty varied across race and ethnicity. 9.79% of White children were in the lowest level of SES status compared to 33.77% of Black and 32.48% of Hispanic children. In addition, 4.83% of White children lived in persistent poverty compared to 21.38% of Black children and 21.30% of Hispanic children. Overall, 13.24% of included cases died from cancer. The most common cause of cancer death was bone tumors (26.44%), CNS tumors (23.06%), soft tissue tumors (22.77%), and hepatic tumors (21.33%) (Figure 2).

**TABLE 1:** Sample demographic characteristics.

Sample characteristics	Total (%) 96,665	Race/ethnicity					*p*
		NH white 46,608 (48.16)	NH black 9955 (10.29)	Hispanic 32,805 (33.90)	AI/AN 514 (0.53)	Asian 6889 (7.12)	
Vital status							
Alive or died of other causes	83,957 (86.76)	41,257 (88.52)	8172 (82.09)	28,147 (85.80)	445 (86.58)	5936 (86.17)	< 0.0001
Died of cancer	12,814 (13.24)	5351 (11.48)	1783 (17.91)	4658 (14.20)	69 (13.42)	953 (13.83)	
Rurality							
Urban	86,891 (89.79)	39,995 (85.81)	9280 (93.22)	30,557 (93.15)	379 (73.74)	6680 (96.97)	< 0.0001
Rural	9880 (10.21)	6613 (14.19)	675 (6.78)	2248 (6.85)	135 (26.26)	209 (3.03)	
Age at diagnosis							
< 1	6474 (6.69)	3061 (6.57)	683 (6.86)	2148 (6.55)	34 (6.61)	548 (7.95)	< 0.0001
1–4	22,852 (23.61)	10,580 (22.70)	2265 (22.74)	8204 (25.01)	136 (26.46)	1667 (24.20)	
5–9	16,887 (17.45)	7832 (16.80)	1738 (17.46)	6038 (18.41)	71 (13.81)	1208 (17.54)	
10–14	19,493 (20.14)	9099 (19.52)	2226 (22.36)	6718 (20.48)	111 (21.60)	1339 (19.44)	
15–19	31,065 (32.10)	16,036 (34.41)	3043 (30.57)	9697 (29.56)	162 (31.52)	2127 (30.88)	
Gender							
Male	51,721 (53.45)	24,795 (53.20)	5151 (51.74)	17,818 (54.31)	255 (49.42)	3703 (53.75)	< 0.0001
Female	45,050 (46.55)	21,813 (46.80)	4804 (48.26)	14,987 (45.69)	260 (50.58)	3186 (46.25)	
SES							
Level 1	19,302 (19.95)	4563 (9.79)	3362 (33.77)	10,665 (32.48)	145 (28.21)	577 (8.38)	< 0.0001
Level 2	17,082 (17.65)	6750 (14.48)	2025 (20.34)	7413 (22.60)	104 (20.23)	790 (11.47)	
Level 3	17,238 (17.81)	8433 (18.09)	1693 (17.01)	6018 (18.34)	99 (19.26)	995 (14.44)	
Level 4	18,946 (19.58)	10,507 (22.54)	1736 (17.44)	5069 (15.45)	85 (16.54)	1549 (22.49)	
Level 5	24,203 (25.01)	16,355 (35.09)	1139 (11.43)	3650 (11.13)	81 (15.76)	2978 (43.23)	
Persistent poverty							
Persistent poverty	11,839 (12.23)	2253 (4.83)	2128 (21.38)	6987 (21.30)	99 (19.26)	372 (5.40)	< 0.0001
Non‐persistent poverty	84,932 (87.77)	44,355 (95.17)	7827 (78.62)	25,818 (78.70)	415 (80.74)	6517 (94.60)	
Cancer type							
Leukemia	26,227 (27.13)	10,812 (23.23)	2216 (22.28)	11,005 (33.57)	169 (32.88)	2025 (29.43)	< 0.0001
CNS	15,988 (16.54)	8713 (18.72)	1741 (17.50)	4500 (13.73)	73 (14.20)	961 (13.97)	
Lymphoma	15,051 (15.57)	7533 (16.19)	1734 (17.43)	4643 (14.16)	56 (10.89)	1085 (15.77)	
Epithelial	10,749 (11.12)	6240 (13.41)	733 (7.37)	2978 (9.08)	59 (11.48)	739 (10.74)	
Germ cell Tumors	6384 (6.60)	2642 (5.68)	473 (4.75)	2703 (8.25)	37 (7.20)	529 (7.69)	
Soft tissue tumors	6268 (6.48)	2902 (6.24)	937 (9.42)	2000 (6.10)	37 (7.20)	392 (5.70)	
Bone tumors	4792 (4.96)	2334 (5.02)	535 (5.38)	1538 (4.69)	24 (4.67)	361 (5.25)	
Neuroblastomas	4158 (4.30)	2271 (4.88)	505 (5.08)	1063 (3.24)	18 (3.50)	301 (4.37)	
Renal Tumors	3438 (3.56)	1602 (3.44)	615 (6.18)	1038 (3.17)	18 (3.50)	165 (2.40)	
Retinoblastomas	1717 (1.78)	693 (1.49)	244 (2.45)	617 (1.88)	12 (2.33)	151 (2.19)	
Hepatic Tumors	1463 (1.51)	602 (1.29)	156 (1.57)	552 (1.68)	8 (1.56)	145 (2.11)	
Others (Unspecified)	430 (0.44)	196 (0.42)	59 (0.59)	145 (0.44)	3 (0.58)	27 (039)	
Stage at diagnosis							
Localized	35,426 (36.65)	18,520 (39.79)	3682 (37.01)	10,725 (32.72)	167 (32.49)	2332 (33.89)	< 0.0001
Regional	16,132 (16.69)	8238 (17.70)	1732 (17.41)	4892 (14.92)	82 (15.95)	1188 (17.26)	
Distant	41,104 (42.52)	17,849 (38.35)	3114 (45.26)	15,842 (48.33)	243 (47.28)	3114 (45.26)	
Unknown/unstaged	4003 (4.14)	1933 (4.15)	478 (4.80)	1323 (4.04)	22 (4.28)	247 (3.59)	

Abbreviations: AI/AN, American Indian/Alaska Native; NH, Non‐Hispanic (SES level 5 is the highest SES group); SES, Socio‐economic Status (SES level 1 is the lowest SES group).

### 3.2 Association of Race and Ethnicity and Socioeconomic Factors With Cancer Mortality

Table 2 presents the association of race and ethnicity and socioeconomic factors (SES and persistent poverty) with pediatric cancer mortality, adjusting for age, rurality, gender, and cancer stage. Compared to White children, the risk of cancer death was higher among Black (aHR: 1.53; 95% CI: 1.45–1.62), Hispanic (aHR: 1.17; 95% CI: 1.12–1.22), and Asian (aHR: 1.24; 95% CI: 1.15–1.33) children. When stratified by cancer type, the hazard of death was consistently higher among Black, Hispanic, and API children compared to White children for all cancer types (Table 3).

**TABLE 2:** Hazard ratio of cancer death by demographic and socio‐economic characteristics.

Characteristics	Univariate analysis		Adjusted analysis	
	HR (95% CI)	*p*	aHR (95% CI)	*p*
Race/ethnicity				
White	Ref			
Black	1.66 (1.57–1.75)	< 0.0001	1.53 (1.45–1.62)	< 0.0001
AI/AN	1.22 (0.96–1.54)	0.1066	1.10 (0.87–1.39)	0.4395
Asian	1.27 (1.19–1.37)	< 0.0001	1.24 (1.15–1.33)	< 0.0001
Hispanics	1.32 (1.27–1.37)	< 0.0001	1.17 (1.12–1.22)	< 0.0001
SES				
Level 1	1.47 (1.40–1.55)	< 0.0001	1.23 (1.15–1.32)	< 0.0001
Level 2	1.32 (1.25–1.40)	< 0.0001	1.20 (1.13–1.27)	< 0.0001
Level 3	1.23 (1.16–1.30)	< 0.0001	1.15 (1.09–1.22)	< 0.0001
Level 4	1.19 (1.12–1.25)	< 0.0001	1.14 (1.08–1.22)	< 0.0001
Level 5	Ref			
Persistent poverty				
Yes, vs. No	1.30 (1.23–1.36)	< 0.0001	1.07 (1.01–1.14)	0.0338

Abbreviations: aHR, Adjusted Hazard Ratio (adjusted for age, gender, rurality and cancer stage); AI/AN, American Indian/Alaska Native; HR, Hazard Ratios; NH, Non‐Hispanic; SES, Socio‐economic Status (SES level 1 is the lowest SES group, SES level 5 is the highest SES group).

**TABLE 3:** Hazard ratio of cancer death by race/ethnicity and cancer types.

Type of cancer	HR (95% CI)			
	Black vs. white	Hispanics vs. white	AI/AN vs. white	Asian vs. white
Leukemia	1.83 (1.64–2.04)*	1.37 (1.27–1.47)*	1.28 (0.85–1.93)	1.21 (1.06–1.38)*
CNS	1.53 (1.38–1.69)*	1.49 (1.39–1.61)*	0.95 (0.57–1.58)	1.36 (1.20–1.55)*
Lymphoma	1.90 (1.56–2.31)*	1.23 (1.04–1.46)*	0.82 (0.21–3.31)	1.17 (0.88–1.57)
Epithelial	2.17 (1.71–2.75)*	1.28 (1.05–1.55)*	1.85 (0.76–4.48)	1.33 (0.98–0.81)
Germ cell Tumors	1.58 (1.1–2.27)*	1.17 (093–1.48)	2.15 (0.80–5.82)	1.33 (0.97–2.07)
Soft tissue tumors	1.12 (0.96–1.31)	1.14 (1.01–1.28)*	0.96 (0.50–1.86)	1.11 (0.89–1.37)
Bone tumors	1.56 (1.31–1.86)*	1.40 (1.24–1.58)*	0.97 (0.44–2.17)	1.14 (0.92–1.43)
Neuroblastomas	1.48 (1.19–1.85)*	1.08 (0.89–1.31)	1.20 (0.38–3.73)	1.21 (0.91–1.62)
Renal Tumors	1.94 (1.42–2.66)*	1.54 (1.15–2.07)*	1.39 (0.19–9.96)	1.86 (1.13–3.12)*
Retinoblastomas	2.21 (0.98–5.12)	1.55 (0.74–3.25)	0.005 (0.0–2.95)	1.58 (0.51–4.89)
Hepatic Tumors	1.32 (0.93–1.87)	0.94 (0.73–1.21)	0.38 (0.053–2.69)	0.85 (0.57–1.28)
Others (Unspecified)	1.99 (0.91–4.36)	1.56 (0.87–2.80)	0.001 (0.0–3.85)	2.11 (0.80–5.60)

* Significant values at 0.05 level, HR: Hazard Ratios, AI/AN: American Indian/Alaska Native. The model is adjusted for age, gender, rurality, and cancer stage.

Socioeconomic characteristics were also associated with cancer mortality (Table 2). Compared to children from higher SES status (level 5), the hazard of death was greater among children from lower SES levels. The adjusted hazard ratios were 1.23 (95% CI: 1.15–1.32), 1.20 (95% CI: 1.13–1.27), 1.15 (95% CI: 1.09–1.22), and 1.14 (95% CI: 1.08–1.22) for SES levels 1, 2, 3, and 4, respectively. Children living in persistent poverty also had a greater adjusted risk of cancer death compared to children living in nonpersistent poverty areas (aHR: 1.07; 95% CI: 1.01–1.14).

### 3.3 Mediation Analysis of Racial and Ethnic Disparity in Childhood Cancer

Table 4 shows the mediation analysis results for all cancer types combined. Using White children as a reference, the mediating effect of SES and persistent poverty was significant for Black, Hispanic, and AI/AN children. SES accounted for 13.50% of the disparity in cancer death for Black, 29.28% for Hispanic, and 37.58% for AI/AN children. The proportion mediated by persistent poverty was 6% for Black, 12.79% for Hispanic, and 17.47% for AI/AN children.

**TABLE 4:** Overall mediating effect of persistent poverty and SES.

Race/ethnicity (vs. white)	Persistent poverty				SES			
	Direct effect (95% CI)	Indirect effect (95% CI)	Total effect (95% CI)	Proportion mediated (%)	Direct effect (95% CI)	Indirect effect (95% CI)	Total effect (95% CI)	Proportion mediated (%)
Black	1.60 (1.51–1.69)*	1.03 (1.02–1.04)*	1.65 (1.54–1.76)*	6.0	1.54 (1.45‐1.63)*	1.07 (1.05–1.09)*	1.65 (1.52–1.77)*	13.50
Hispanics	1.22 (1.17–1.27)*	1.03 (1.02–1.04)*	1.26 (1.19–1.32)*	12.79	1.18 (1.12‐1.23)*	1.07 (1.05–1.09)*	1.26 (1.20–1.34)*	29.28
AI/AN	1.10 (0.86–1.39)	1.02 (1.01–1.03)*	1.12 (0.87–1.43)	17.47	1.07 (0.84‐1.36)	1.04 (1.03–1.06)*	1.11 (0.87‐1.44)	37.58
Asian	1.22 (1.14–1.32)*	1.002 (1.0–1.003)	1.22 (1.14–1.32)*	1.0	1.24 (1.14‐1.34)*	1.0 (0.99–1.0)	1.24 (1.12–1.34)*	0

* Significant values, AI/AN: American Indian/Alaska Native. The direct, indirect, and total effects are on a hazard ratio scale.

Tables 5 and 6 show the mediating effects of persistent poverty and SES for the top three common cancers (leukemia, CNS tumors, and lymphoma), respectively. Compared to White children, among children diagnosed with leukemia, SES explained 32.57% of disparities in mortality for Hispanic, 26.76% for AI/AN, and 16.24% for Black children. For CNS tumors, the proportion mediated was 12.23% for Hispanic and 13.70% for Black children. For lymphoma, the proportion mediated was 66.47% for Hispanic, 19.36% for Black, and 61.46% for Asian children compared to White children (Table 5). The mediating effect of persistent poverty was significant only for leukemia and CNS tumors. For leukemia, persistent poverty explained 9.61% of differences in mortality between Hispanic and White children, 5.76% for Black children, and 10.39% for AI/AN children compared to White children. Among children diagnosed with CNS tumors, persistent poverty accounted for 7.41% of mortality differences for Hispanic compared to White children and 7.17% for Black when compared to White children. The mediating effect of persistent poverty was not significant for all races among children diagnosed with lymphoma (Table 6).

**TABLE 5:** Mediating effect of ses by cancer types.

Cancer type	Race/ethnicity (vs. white)	SES			
		Direct effect (95% CI)	Indirect effect (95% CI)	Total effect (95% CI)	Proportion mediated (%)
Leukemia	Black	1.56 (1.39–1.78)*	1.09 (1.06–1.13)*	1.70 (1.47–2.01)*	16.24
	Hispanics	1.22 (1.12–1.33)*	1.10 (1.06–1.14)*	1.34 (1.19–1.52)*	32.57
	AI/AN	1.14 (0.75–1.71)	1.05 (1.023–1.08)*	1.20 (0.77–1.85)	26.76
	Asian	1.17 (0.999–1.37)	0.99 (0.98–1.0)	1.16 (0.98–1.37)	−6.77
CNS	Black	1.44 (1.29–1.61)*	1.06 (1.03–1.09)*	1.53 (1.33–1.75)*	13.70
	Hispanics	1.42 (1.31–1.54)*	1.05 (1.02–1.09)*	1.49 (1.34–1.68)*	12.23
	AI/AN	0.88 (0.53–1.48)	1.05 (1.01–1.08)*	0.88 (0.54–1.60)	−38.17
	Asian	1.52 (1.30–1.76)*	0.99 (0.97–1.001)	1.50 (1.26–1.76)*	−2.48
Lymphoma	Black	1.66 (1.34–2.06)*	1.13 (1.06–1.21)*	1.88 (1.42–2.49)*	19.36
	Hispanics	1.05 (0.87–1.27)	1.11 (1.03–1.20)*	1.17 (0.90–1.52)	66.47
	AI/AN	0.59 (0.15–2.26)	0.99 (0.97–1.004)	0.58 (0.15–2.27)	1.85
	Asian	1.08 (0.78–1.49)	1.13 (1.06–1.21) *	1.22 (0.83–1.80)	61.46

* Significant values, AI/AN: American Indian/Alaska Native. The direct, indirect, and total effects are on a hazard ratio scale.

**TABLE 6:** Mediating effect of persistent poverty by cancer types.

Cancer type	Race/Ethnicity (vs. white)	Persistent poverty			
		Direct effect (95% CI)	Indirect effect (95% CI)	Total effect (95% CI)	Proportion mediated (%)
Leukemia	Black	1.62 (1.44–1.82)*	1.03 (1.004–1.05)*	1.67 (1.45–1.91)*	5.76
	Hispanics	1.32 (1.22–1.43)*	1.03 (1.005–1.05)*	1.36 (1.23–1.50)*	9.61
	AI/AN	1.19 (0.79–1.81)	1.02 (1.001–1.034)*	1.21 (0.79–1.87)	10.39
	Asian	1.18 (1.02–1.36)*	1.001 (0.999–1.003)	1.18 (1.02–1.36)*	0.6
CNS	Black	1.47 (1.32–1.63)*	1.03 (1.01–1.06)*	1.51 (1.33–1.73)*	7.17
	Hispanics	1.45 (1.34–1.56)*	1.03 (1.01–1.05)*	1.49 (1.35–1.64)*	7.41
	AI/AN	1.04 (0.61–1.77)	1.02 (0.999–1.04)	1.06 (0.61–1.84)	33.98
	Asian	1.44 (1.25–1.65)*	1.002 (0.999–1.01)	1.44 (1.25–1.67)*	0.55
Lymphoma	Black	1.92 (1.56–2.36)*	1.0001 (0.96–1.05)	1.92 (1.50–2.45)*	0.02
	Hispanics	1.20 (1.01–1.44)*	1.0001 (0.96–1.04)	1.20 (0.97–1.50)	0.05
	AI/AN	0.59 (0.16–2.19)	1.00 (0.96–1.04)	0.59 (0.15–2.28)	0
	Asian	1.19 (0.87–1.63)	1.0 (0.99–1.005)	1.19 (0.86–1.64)	0

* Significant values, AI/AN: American Indian/Alaska Native. The direct, indirect, and total effects are on a hazard ratio scale.

## 4 Discussion

This study used SEER Research Plus Specialized Incidence Data with the Census Tract Attributes Database to examine the racial and ethnic disparities in childhood cancer‐specific mortality and the role of SES and persistent poverty in those disparities. Overall, we found that compared to White children, the hazard of dying from cancer was higher among Black, Hispanic, and Asian children. The mediation analysis highlighted the role of socioeconomic factors in racial and ethnic disparities in childhood cancer mortality, underscoring the need for interventions targeting SES factors to address racial and ethnic disparities in childhood cancer outcomes. The proportion mediated was significant for both persistent poverty and SES among Black, Hispanic, and AI/AN compared to White children. However, the proportion mediated was higher for SES than for persistent poverty, which may be explained by two main differences in how these variables are operationalized. First, SES is a single time‐point measure indicating SES at the child's cancer diagnosis. On the other hand, persistent poverty reflects the long‐lasting neighborhood poverty. Second, SES is a composite variable encompassing various socioeconomic factors (including the proportion employed in working‐class occupations, the proportion aged 16 years or older and unemployed, education index, median household income, proportion below the 200% poverty level, median rent, and median house income). On the other hand, persistent poverty is a binary measure that indicates whether a neighborhood has 20% or more of its population living below the poverty level for approximately 30 years or more. This provides less variability across socioeconomic factors integrated into the measure, likely resulting in a low proportion mediated in our study results. In addition to differences in variable measurements, neighborhood SES likely plays a more significant role as a mediator than persistent poverty, as indicated by the higher proportion mediated. SES reflects the current socioeconomic challenges that can hinder access to healthcare and the ability to navigate treatment during the diagnosis and treatment phases, ultimately influencing mortality outcomes. In contrast, persistent poverty affects the underlying socioeconomic structures and trajectories over the long term, which are later reflected in SES at the time of diagnosis. This indicates that interventions aimed at improving SES can lead to quick and substantial reductions in racial and ethnic disparities in pediatric cancer mortality.

Our results are consistent with previous studies indicating racial and ethnic disparities in pediatric cancer mortality [3, 21, 32, 33, 34, 35]. White children consistently have better survival outcomes compared to minority races, even after adjusting for cancer stage, treatment received, age, and gender [36, 37]. The results have been the same across different cancers such as lymphoma, CNS tumors, leukemia, and Wilms' tumors [6]. Various factors, including poor prognosis, late stage at diagnosis, and underrepresentation in clinical trials, are thought to contribute to racial and ethnic differences in childhood cancer mortality [38]. In addition, some studies have shown that minority races experience access to care issues such as limited transportation, higher healthcare costs, and greater distance to the hospital, which could exacerbate racial and ethnic disparities in childhood cancer mortality [22, 38, 39, 40].

Neighborhood SES was evaluated as a potential mediator in this study. Consistent with previous studies conducted among adults [41, 42], our mediation analysis results showed SES as a pathway that could explain racial ethnic disparities in childhood cancer mortality. SES mediated the effects of race and ethnicity on survival, mainly for Black and Hispanic [41, 42]. Our results are consistent with one study conducted among pediatric cancer population [23]. However, in this study, the proportion mediated for different cancer types was significantly higher than the results from our study. For example, the proportion for Black vs. White children was 44% for acute lymphoblastic leukemia and 28% for acute myeloid leukemia, while in our study, it was 16.24% for leukemia [23]. This difference could be explained by the cancer classification choices used in both studies. Although both studies used ICCC‐3, our study estimated the proportion mediated using major groupings of cancer types (e.g., leukemia, lymphoma, CNS tumors), while the previous study assessed mediation by more detailed sub‐types (e.g., acute lymphoblastic leukemia, acute myeloid leukemia, non‐Hodgkin lymphoma, Hodgkin lymphoma, astrocytoma, and non‐astrocytoma CNS tumors). Grouping cancer types into broader categories may decrease or mask the effect for specific subtypes, leading to a lower observed proportion mediated.

Persistent poverty was also a significant mediator of race and ethnicity and pediatric cancer mortality in our study. While this study is the first to examine persistent poverty as a mediating factor in racial and ethnic mortality disparities of childhood cancers, existing evidence assessing the effect of persistent poverty on cancer survival indicates that children living in persistent poverty areas have poor survival [12]. Children living in disadvantaged neighborhoods may have difficult access to healthcare and are more likely to be diagnosed at a later stage, thus having worse survival outcomes [43]. Furthermore, children from neighborhoods with persistent poverty may experience food insecurity, housing instability, and financial hardship, all of which will contribute to worse survival outcomes [14, 44, 45].

The effect of persistent poverty on pediatric cancer survival also varied by cancer types in our study. The proportion mediated by persistent poverty was significant only for Black, AI/AN, and Hispanic children compared to White children for leukemia and only among Hispanic and Black children with CNS tumors. Previous studies indicate that the effect of persistent poverty varies across different types of cancer [12]. For example, Hymel et al. reported that persistent poverty was associated with higher mortality among children with leukemia, CNS, and hepatic tumors [12]. The variation in tumor biology, clinical presentations, and treatment intensity across cancer types and race may contribute to observed differences in survival outcomes [46], potentially leading to observed differential effects of persistent poverty across cancer types. Future studies should evaluate the interplay of persistent poverty and tumor‐related factors across racial and ethnic groups.

### 4.1 Study Implications

The study findings highlight the role of neighborhood‐level SES and persistent poverty in racial and ethnic disparities in pediatric cancer mortality. These results may inform risk classification, enabling clinicians to identify children at risk of poor survival due to neighborhood disadvantages and implement targeted interventions. Such interventions may include referrals to housing programs, food, and transportation support, enhanced social work services, improving communication, and reducing language barriers [46, 47, 48]. Previous studies have also commended integrating support models of care that consider social risk and enhanced navigation programs for the minority population [46, 47]. Furthermore, given the long‐lasting impact of socioeconomic factors such as persistent poverty, policies that address the adverse social and environmental conditions in addition to income and wealth inequality could reduce disparities in cancer outcomes [47]. To close the racial and ethnic gap in pediatric cancer survival outcomes, a multi‐level approach combining policy and targeted individual and neighborhood‐level interventions is warranted.

### 4.2 Limitations

This study has some limitations. First, individual SES information is not available within SEER; therefore, we used area‐based socioeconomic factor variables and were unable to explore the role of individual socioeconomic factors. Moreover, SES and persistent poverty classification are based on the address at diagnosis. The lack of a complete address at diagnosis may lead to misclassification. Second, although racial and ethnic disparities in pediatric cancer mortality could be explained by other individual factors, such as biological factors and access to care, we could not explore those factors as they are not available in the SEER database. Additionally, clinical factors such as tumor biology and treatment received were not accounted for in our analysis. Third, the cause of death in the SEER database is extracted from death certificates. Hence, there is a potential for misclassification. Fourth, when doing mediation analysis for the top three cancer types, the sample size for Asian and AI/AN children may have been too small to identify the significant contribution of socioeconomic factors in racial and ethnic differences in mortality. Furthermore, we limited the moderated mediation to only the three most common cancers due to sample size limitations. Hence, studies examining the mediating effect of socioeconomic factors in other types of cancers are warranted. Nevertheless, the study provides an understanding of the contribution of SES and persistent poverty in racial and ethnic disparities in pediatric cancer mortality using a population‐based study and a large sample size.

## 5 Conclusion

Consistent with previous studies, this study showed significant racial and ethnic disparities in pediatric cancer mortality. Neighborhood SES and persistent poverty account for a significant proportion of those disparities. Although the proportion mediated by persistent poverty was small (< 10%) among Black and Asian children, it was statistically significant overall and among children with leukemia and CNS tumors, highlighting the long‐lasting effect of neighborhood disadvantage on pediatric cancer survival. The proportion mediated by SES was also significant for leukemia, CNS tumors, and lymphoma, indicating that interventions addressing those socioeconomic factors can reduce mortality disparities for those types of cancers. The study findings highlight the need for a comprehensive intervention approach that targets individual and neighborhood‐level socioeconomic factors that hinder access to care and treatment adherence. Such interventions may reduce childhood cancer mortality disparities.

## Author Contributions

**Josiane Kabayundo:** conceptualization (equal), data curation (lead), formal analysis (lead), methodology (equal), software (equal), visualization (lead), writing – original draft (lead), writing – review and editing (equal). **Apu Das:** conceptualization (equal), formal analysis (equal), methodology (equal), software (equal), validation (equal), writing – review and editing (equal). **Cheng Zheng:** conceptualization (equal), methodology (equal), supervision (equal), writing – review and editing (equal). **Emma Hymel:** conceptualization (equal), methodology (equal), writing – review and editing (equal). **Krishtee Napit:** conceptualization (equal), methodology (equal), writing – review and editing (equal). **Shinobu Watanabe‐Galloway:** conceptualization (equal), methodology (equal), supervision (lead), writing – review and editing (equal).

## Funding

The authors have nothing to report.

## Ethics Statement

This study was conducted using de‐identified, publicly available data from the Surveillance, Epidemiology, and End Results Program; hence, it did not require Institutional Review Board approval or informed consent.

## Conflicts of Interest

The authors declare no conflicts of interest.

## Data Availability

The data used in this study are publicly available from the US National Cancer Institute's Surveillance, Epidemiology, and End Results (SEER) Program. The data can be requested at https://seer.cancer.gov/data/specialized/available‐databases/census‐tract‐request/.
